# Genetic Polymorphisms Associated with Environmental Exposure to Polycyclic Derivatives in African Children

**DOI:** 10.1155/2018/9078939

**Published:** 2018-08-01

**Authors:** Rodrigo Mota de Oliveira, Camylla Vilas Boas Figueiredo, Rayra Pereira Santiago, Sètondji Cocou Modeste Alexandre Yahouédéhou, Suéllen Pinheiro Carvalho, Silvana Souza da Paz, Luciana Magalhães Fiuza, Fernando Nunes de Miranda, Caroline Conceição da Guarda, Cleverson Alves Fonseca, Milena Magalhães Aleluia, Cynara Gomes Barbosa, Elisângela Vitória Adorno, Marilda de Souza Gonçalves

**Affiliations:** ^1^Laboratório de Pesquisa em Anemia (LPA), Departamento de Análises Clínicas e Toxicológicas, Faculdade de Farmácia, Universidade Federal da Bahia, Rua Barão do Jeremoabo, no. 147, Ondina, 40.170-115 Salvador, BA, Brazil; ^2^Laboratório de Investigação em Genética e Hematologia Translacional (LIGHT), Instituto Gonçalo Moniz (IGM), FIOCRUZ, Rua Waldemar Falcão, 121 Candeal, 40.296-710 Salvador, BA, Brazil

## Abstract

**Background:**

The nonracial leukopenia may be a result of exposure to polycyclic derivatives (benzene-toluene-xylene (BTX)) and may arise from a possible change in the bone marrow microenvironment. The present study sought to evaluate the association of genetic polymorphisms in xenobiotic-metabolizing enzymes with hematological and biochemical profiles.

**Methods:**

We evaluated 89 African descendant children, exposed indirectly to benzene derivatives. Laboratory parameters were investigated by automated methods and genetic polymorphisms by PCR-RFLP and PCR multiplex.

**Results:**

Children with leukopenia had significantly decreased white blood cells (WBCs) and platelet counts, which is not consistent with benign leukopenia. In the same group, we have found that carriers of the *CYP2E1* variant allele had decreased WBC and lymphocytes. Those with *NQO1* variant allele had decreased WBC, neutrophil, eosinophil, monocyte, and lymphocyte counts. Carriers of the *MPO* variant allele had decreased WBC, neutrophil, eosinophil, basophil, monocyte, lymphocyte, and platelet counts and an elevated free iron level. Children with *GSTT* and *GSTM* null exhibited decreased WBC, neutrophil, basophil, and lymphocyte counts. Our multivariate analysis model reveals that females were independently associated with leukopenia.

**Conclusion:**

Our results suggest that the polymorphisms investigated were associated with hematological changes in the studied population. These alterations could be heightened by exposure to benzene derivatives.

## 1. Introduction

Xenobiotic compounds are classified as any foreign chemical substance inside the biological system. Most xenobiotics that the humans are exposed come from environmental pollution, food additives, cosmetics, agricultural products, toxic agents, and drugs. Usually xenobiotics are lipophilic, and if they do not undergo regular metabolism, they can be potentially harmful to exposed humans. Under physiological conditions, humans exhibit mechanisms responsible for enzymatic metabolism or biotransformation of xenobiotics. This involves the biotransformation based on phase I and phase II reactions. During the first phase, the oxidation and reduction of hydrophobic chemicals occur, while in the second phase the conjugation reactions (acetylation, methylation, and glucuronidation) take place in order to remove the byproducts from the human organism as urine or sweat [1, 2].

Human exposure to refining and petroleum refinery process derivatives can happen indirectly in the environment. These derivatives are able to promote substantial changes in human health. Polycyclic aromatic derivative causes hematological abnormalities, such as leukopenia, eosinophilia, and thrombocytopenia. The benzene-toluene-xylene fraction (BTX) changes the bone marrow microenvironment, inhibiting the hematopoiesis [3]. In addition, hematological disorders may also be related to polymorphisms in genes encoding xenobiotic-metabolizing enzymes, such as cytochrome P4502E1 (CYP2E1), myeloperoxidase (MPO), NAD(P)H:quinone oxidoreductase 1 (NQO1), and glutathione S-transferase (GST) [3–6].

The CYP2E1 is an enzyme responsible for the metabolism of xenobiotics, including toxic and therapeutic agents, and is involved in oxidative bioactivation of hydrophobic chemicals, such as benzene and acrylamide. *CYP2E1 -1293G>A/-1053C>T* polymorphisms are associated to increased risk of developing acute myeloid leukemia (AML) and acute lymphocytic leukemia (ALL) [7, 8].

NQO1 is an enzyme involved in regulating and reducing chemical compounds that have quinone in their structure. This enzyme prevents the production of free oxygen radicals, thus protecting the cell from oxidative stress. *NQO1 609C>T* single nucleotide polymorphism (SNP) is associated with decreased enzyme activity and an increased susceptibility to develop leukemia and bladder cancer [9, 10].

MPO is an oxidizing enzyme, usually restricted to myeloid cells. The enzyme is involved in the biotransformation of cigarette smoke in the highly reactive and carcinogenic benzene intermediate. This enzyme is abundant in neutrophils and monocytes and catalyzes hydrogen peroxide (H_2_O_2_) to hypochlorous acid (HOCl), a potent oxidant. The polymorphism *MPO -463G>A* leads to enzymatic changes, affecting the metabolism of xenobiotics, which is related to the formation of DNA adducts [3, 11–13].

GST comprises a family of 16 genes involved in six subfamilies: *α* (GSTA), *μ* (GSTM), *Ω* (GSTO), *π* (GSTP), *θ* (GSTT), and *ζ* (GSTZ), and they play an important role in the cellular detoxification of chemical compounds both from endogenous and exogenous origins. GSTM1 and GSTT1 catalyze the conjugation reaction of glutathione with hydrophobic chemicals. A large deletion of the whole gene in homozygous (*GSTM1*^∗^*0* null alleles or *GSTT1*^∗^*0*) results in insufficient enzyme activity, while the heterozygotes present reduced enzyme activity [14–16].

São Francisco do Conde (SFC) is a city with African origin or quilombola, a designation given to the remaining populations of quilombos, which are descendants of black slaves who preserve characteristics of the original African population [17]. In the same geographical area, an oil industry is present, which is responsible for processing and refining petroleum, which can deliver volatile organic compounds daily to the atmosphere, derived from oil [18]. In African descendant populations, ethnic-related hematological changes are present, although without clinical relevance. However, once these hematological changes are associated with environment exposure, significant characteristics may be prominent.

Thus, the aim of this study was to investigate the association of variant alleles in genes encoding xenobiotic-metabolizing enzymes, with the hematological profiles of individuals who exhibited leukopenia.

## 2. Materials and Methods

### 2.1. Subjects

The present cross sectional study included 89 children with median age of 9 years, aged 6–12 years, all resident in the quilombola communities located in São Francisco do Conde (SFC), Bahia, Brazil, from October 2010 to March 2011. These communities are located approximately 4.47 miles far from the oil industry.

We identified thirty-two children with leukopenia, that is, white blood cell (WBC) counts below 4.0 × 10^3^/mL, followed by peripheral neutropenia (neutrophils < 1.5 × 10^3^/mL), whereas 57 children with normal hematological parameters were matched as control group by age and the same geographic origin.

Since all individuals were under 18 years, their legal guardians agreed to biological sample collection procedures and signed terms of informed consent were provided. The study was approved by the Research Board of the Instituto Gonçalo Moniz, Fundação Oswaldo Cruz (IGM-FIOCRUZ) and was conducted in compliance with ethical principles of the Declaration of Helsinki as well as its revision.

### 2.2. Socioeconomic Characteristics

The socioeconomic characteristics of the SFC population are quite contradictory, since the city has one of the largest gross domestic product and still has an elevated rate of poverty. The surveyed community has very low family income, poor basic sanitation, exposure to toxic substances, and infections. Many parents of the children we evaluated only went to elementary school, which contributes to the informal employment; hence, they have a very low purchasing power and are included in assistance programs of government in order to increase the family income.

### 2.3. Samples

Venous blood samples were collected by venipuncture the morning after 12 h of fasting in standardized conditions, at enrolment, by a specialized professional. One EDTA tube was collected to perform the hematological and genetic analysis and one tube without additives for the accomplishment of the biochemical measurement. The venous blood collection was performed in the quilombola communities of SFC, and the material was sent to the Department of Clinical Analyses and Toxicology located at Faculdade de Farmácia at Universidade Federal da Bahia (UFBA) for hematological, biochemical, and genetic analysis.

### 2.4. Hematological and Biochemical Data

Blood counts were performed in an automated machine (CELL-DYN Ruby, Abbott Diagnostics, Illinois, USA) at Faculdade de Farmácia at Universidade Federal da Bahia (UFBA). Biochemical analyses (uric acid, total cholesterol and fractions, creatinine, free iron, glucose, and urea) were performed on equipment COBAS INTEGRA 400 plus (Indianapolis, Indiana, USA), according to the manufacturer's instructions.

### 2.5. Molecular Analysis

DNA extraction was performed in peripheral blood leukocytes using DNA Kit 250 FlexiGene Qiagen (Hilden, North Rhine-Westphalia, Germany) in the Laboratório de Investigação em Genética e Hematologia Translacional (LIGHT) of the IGM-FIOCRUZ. Evaluation of *CYP2E1 -1293G>C/-1053C>T* (rs3813867/rs2031920), *NQO1 609C>T* (rs1800566), and *MPO -463G>A* (rs2333227) gene polymorphisms were performed by PCR/RFLP (polymerase chain reaction/restriction fragment length polymorphism) [10, 11, 19]. Analysis of *GSTT1*/*GSTM1* gene polymorphisms was performed by multiplex PCR, using the *HBB* (beta-globin) gene as an endogenous marker for the reaction [14].

### 2.6. Statistical Analysis

Statistical analyses were performed using the Statistical Package for the Social Sciences (SPSS) version 21.0 and GraphPad version 7.0. We considered significant *p* values < 0.05. Analysis of normal distribution of quantitative variables was performed using the Kolmogorov-Smirnov test and the independent *t*-test, and Mann–Whitney *U* test was used for analyses of two variables comparing two groups of values within a given variable, according to the distribution of each variable. Multivariate binary logistic regression analysis was employed to assess the goodness of fit of a model designed to evaluate possible associations between leukopenia and a group of other characteristics.

## 3. Results

The baseline characteristics of the 32 leukopenic and 57 controls enrolled children including mean ± standard deviation of laboratory parameters are shown in Table 1.

**Figure 1:**
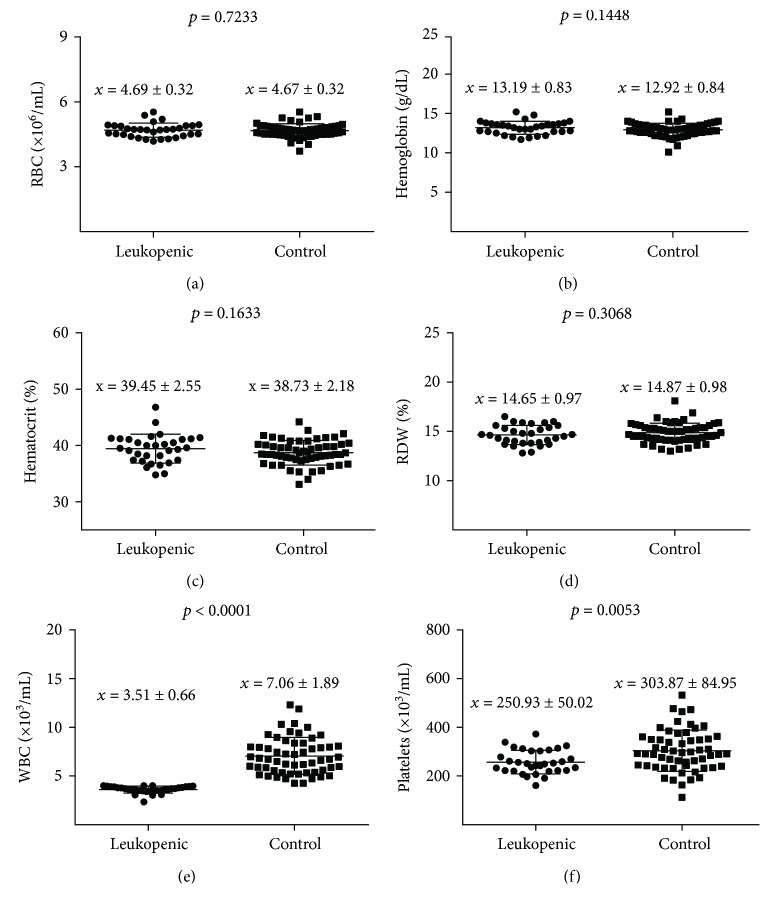
Comparison of hematological parameters between leukopenic group and controls ((a) RBC; (b) hemoglobin; (c) hematocrit; (d) RDW; (e) WBC, and (f) platelets).

A comparison of the hematological and biochemical parameters between children with leukopenia and the control group found significantly decreased WBC and platelet counts in leukopenic children (Figure 1). No significant differences were found regarding red blood cell counts.

### 3.1. Association of the CYP2E1 Variant Allele c2 with Hematological and Biochemical Profiles

In order to investigate the association between CYP2E1 variant allele c2 and hematological and biochemical parameters, we selected in the leukopenic and control groups individuals with variant allele c2. WBC (*p* = 0.004) and lymphocyte counts were found to be significantly decreased in children with leukopenia (Table 2).

### 3.2. Association of the NQO1 Variant Allele T with Hematological and Biochemical Profiles

The association of *NQO1* variant allele T with hematological and biochemical characteristics was also investigated. Among children with leukopenia, we found decreased WBC (*p* = 0.0001), neutrophils (*p* = 0.0001), eosinophils (*p* = 0.005), monocytes (*p* = 0.002), and lymphocytes (*p* = 0.0001) (Table 2).

### 3.3. Association of the MPO Variant Allele A with Hematological and Biochemical Profiles

Children with leukopenia carriers of the variant allele A for MPO gene polymorphism also had significantly decreased WBC (*p* = 0.0001), neutrophils (*p* = 0.0001), eosinophils (*p* = 0.032), basophils (*p* = 0.002), monocytes (*p* = 0.0007), lymphocytes (*p* = 0.0001), and platelets (*p* = 0.004); in addition, we also found an elevated free iron level (*p* = 0.018) (Table 3).

### 3.4. Association of the GSTT1 Null with Hematological and Biochemical Profiles

The association of GSTT1 null gene polymorphism was also tested among children with leukopenia. We found significantly decreased WBC (*p* = 0.0021), neutrophil (*p* = 0.0001), basophil (*p* = 0.002), and lymphocyte (*p* = 0.014) counts (Table 4).

### 3.5. Association of the GSTM1 Null with Hematological and Biochemical Profiles

A comparison of GSTM1 null gene polymorphism in the group of children with leukopenia also found to be significantly decreased WBC (*p* = 0.0001), neutrophil (*p* = 0.0001), basophil (*p* = 0.0449), lymphocyte (*p* = 0.0001), and monocyte (*p* = 0.0014) counts (Table 4).

### 3.6. Multivariate Analysis Model

Our three multivariate analysis model (*p* = 0.049, *p* = 0.037, and *p* = 0.048) were designed to investigate any associations between polymorphisms, age, and sex with leukopenia (Table 5). From all the variables employed in the models, we found that females were independently associated with leukopenia.

## 4. Discussion

The decrease in the WBC counts verified herein in the leukopenic group may be related to racial variation as well as the presence of an underlying pathology. However, in the racial variation, also named benign leukopenia, the neutrophil count is never as low as 1.5 × 10^3^/mL. Individuals with racial neutropenia do not exhibit significant changes in WBC counts, and variations in cell morphology may be present [20–22]. To date, no data was published regarding the association between ethnicity and decreased platelet counts, as observed in our group of children who had leukopenia. Thus, we hypothesized that the leukopenia that children exhibited was not a benign leukopenia and may be increased by the environmental exposure to petroleum derivatives.

The BTX fraction of petroleum alters the bone marrow microenvironment, inhibiting hematopoiesis. Neutropenia associated with the toxicity of chemicals is related to polycyclic aromatic part of benzene derivatives. Decreased WBC counts followed by neutropenia correspond to the main hematologic effect of benzene secondary to hypoplasia and, less frequently, only thrombocytopenia or associated with neutropenia [5, 23]. Decreased counts of WBC, neutrophils, lymphocytes, and monocytes were found in the recent study designed to evaluate the hematotoxicity of low-level benzene exposure among workers from a petrochemical factory [24]. Similarly, in an evaluation among pesticide retailers, the results of hematological assessment showed that monocyte counts, hemoglobin, hematocrit, mean corpuscular volume, mean corpuscular hemoglobin, and platelet distribution width levels were also significantly lower [25]. Altogether, these results suggest that occupational exposure may be responsible for the hematological changes exhibited by the workers.

The toxic effect of benzoquinone occurs due to the formation of DNA adducts and the production of reactive oxygen species (ROS). The benzene toxicity affects the bone marrow stroma cells, which hinders the production of physiological levels of cytokines which are responsible for maintaining both growth and development of hematopoietic cells. Exposure to benzene and its derivatives may promote a decrease in hemoglobin concentrations, red blood cell (RBC) counts, WBC counts, and progenitor cells. The absorption of the toluene fraction has been associated with bone marrow damage and impaired hematopoiesis, as observed in animal models; however, more studies are needed to clarify the underlying mechanism [5, 23, 26]. A description of two cases has also verified complex chromosomal rearrangements and lower natural killer (NK) cells in female Brazilian gas station workers who presented benzene poisoning symptoms and miscarriage history [27].

Carrier individuals of the variant allele of *CYP2E1 -1293G>C/-1053C>T* (rs3813867/rs2031920) who are also exposed to polycyclic aromatic derivatives may have a high CYP2E1 activity and are prone to develop hematological malignancies, such as AML and ALL. The presence of the variant allele of these SNPs alters enzymatic activity, affecting the metabolism pathway; hence, the elimination of toxic substances from the human body is also impaired. Herein, the presence of the variant allele of *CYP2E1 -1293G>C/-1053C>T* (rs3813867/rs2031920) was associated with decreased WBC counts. This effect may be explained by the population ethnic origin, which is also heightened by the exposure to chemicals, which affect the production of the blood cells [8]. The evaluation of variant allele of *CYP2E1* in sickle cell anemia patients also suggested an association with hematological changes [28].

In this study, we observed that WBC counts were reduced in carrier children of the variant allele in *NQO1 609C>T* (rs1800566). Studies suggest that the polymorphism in *NQO1 609C>T* (rs1800566) is related to toxicity of benzene, and this, in turn, is associated with damage in the bone marrow, since its metabolites are topoisomerase II inhibitors. Carrier individuals of the variant allele in *NQO1 609C>T* (rs1800566) that have a null or reduced activity of the enzyme have increased risk for leukemia. The NQO1 enzyme is involved in the metabolism of xenobiotics, which confers protection against cancers, since this enzyme can stabilize *p53* and, thus, assists the apoptosis, as well as the clonal expansion of malignant cells. The decreased or null NQO1 activity is due to polymorphisms which reduce or eliminate these benefits and thus contributes to the incidence of cancer [29, 30].

In the present study, we observed that the *MPO -463G>A* (rs2333227) SNP was significantly associated in the leukopenic group to decreased WBC and platelet counts. The *MPO -463G>A* (rs2333227) polymorphism may result in a decreased enzyme activity and even greater association with AML, since homozygotes for the G allele correspond to 80% of cases of acute promyelocytic leukemia. We also found that the presence of the variant allele in the *MPO -463G>A* (rs2333227) was associated with increased free iron levels in the leukopenic group, which may be related with increased oxidative stress, whereas transition metals may donate or accept electrons during intracellular responses by promoting production of free radicals [29, 31, 32].

Deletions in *GSTM1* and *GSTT1* genes are associated with reduced or null enzyme activity. Studies have shown that there is an association between the decreased enzyme activity and the susceptibility to develop several types of cancer, such as oral, gastric, and bladder cancer, as well as chronic myeloid leukemia (CML) in different ethnic groups worldwide [33, 34]. The WBC and neutrophil counts were decreased in the leukopenic group. This finding may be suggestive of hematological disorders of the bone marrow microenvironment in these children, leading to a possible peripheral pancytopenia, increased by the exposure to benzene derivatives. The presence of the *GSTM1* null genotype may increase the risk to develop AML, as observed in an East Asian population, while the total deletion in *GSTT1* increases the risk of AML in Asian Caucasians. The double deletion in these genes confers a risk of 5.92 times greater to the development of leukopenia in the Brazilian population, which may progress to pancytopenia or cell aplasia [14, 35].

Our multivariate analysis shows that female gender and age (older than 9 years) are suggestive as protective factors for the development of leukopenia, even with the polymorphisms in *NQO1 609C>T* or *GSTM1*. However, when we evaluate *NQO1 609C>T* and *GSTM1* gene polymorphisms, the occurrence of leukopenia is favored (model 3, Table 5). This can be explained by the fact that male children younger than 9 years old follow their mothers at work, since many women are responsible for the financial support of the home, through fishing in the geographical region of SFC.

The present study allowed us to identify the hematological profile of individuals with leukopenia and associate these parameters with polymorphisms in genes encoding xenobiotic-metabolizing enzymes. However, our study design does not allow the long-term follow-up of individuals exposed to petroleum derivatives to determine whether these hematological changes will eventually lead to the development of a malignant disease. Still, it is important to highlight that the genetic sensitivity to the environment exposure is a complex association and a constant hematological evaluation is useful to monitor this possible outcome.

## 5. Conclusion

This study showed that *MPO -463G>A* (rs2333227), *CYP2E1 -1293G>C/-1053C>T* (rs3813867/rs2031920), *NQO1 609C>T* (rs1800566), *GSTT1*, and *GSTM1* gene polymorphisms are associated with hematological alterations. This population, specifically, may have these biomarkers enhanced through the exposure to polycyclic aromatic derivatives, since there was also a reduction in neutrophil and platelet counts.

## Figures and Tables

**Table 1:** Comparison of hematological characteristics between leukopenic and control children.

Hematological parameters	Leukopenic (32)	Controls (57)
	Mean ± SD	Mean ± SD
RBC (×10^6^/mL)	4.69 ± 0.32	4.67 ± 0.32
Hemoglobin (g/dL)	13.19 ± 0.83	12.92 ± 0.83
Hematocrit (%)	39.45 ± 2.55	38.73 ± 2.55
MCV	84.15 ± 3.57	83.14 ± 3.57
MCH	28.02 ± 1.65	27.75 ± 1.65
CHCM	33.29 ± 1.22	33.36 ± 1.22
RDW (%)	14.65 ± 0.97	14.87 ± 0.97
WBC (×10^3^/mL)	3.51 ± 0.66	7.06 ± 0.66
Neutrophils (×10^3^/mL)	1.13 ± 0.34	3.08 ± 0.34
Eosinophils (×10^3^/mL)	0.23 ± 0.15	0.52 ± 0.15
Basophils (×10^3^/mL)	0.05 ± 0.03	0.08 ± 0.03
Lymphocytes (×10^3^/mL)	1.80 ± 0.38	2.88 ± 0.38
Monocytes (×10^3^/mL)	0.31 ± 0.08	0.49 ± 0.08
Platelets (×10^3^/mL)	250.93 ± 59.02	303.87 ± 59.02

SD: standard deviation; RBC: red blood cell; MCV: mean corpuscular volume; MCH: mean corpuscular hemoglobin; MCHC: mean corpuscular hemoglobin concentration; RDW: red cell distribution width; WBC: white blood cell; HDL-c: high-density lipoproteins of cholesterol; LDL-c: low-density lipoproteins of cholesterol.

**Table 2:** Association of hematological parameters with variant allele of *CYP2E1 -1293G>C/-1053C>T* and *NQO1 609C>T* polymorphisms in both leukopenic and control children.

Hematological parameters	Presence of the *CYP2E1* variant allele c2			Presence of the *NQO1* variant allele T		
	Leukopenic (3)	Controls (6)	*p*	Leukopenic (17)	Controls (26)	*p*
	Mean ± SD	Mean ± SD		Mean ± SD	Mean ± SD	
RBC (×10^6^/mL)	4.56 ± 0.09	4.59 ± 0.07	0.7843	4.67 ± 0.09	4.57 ± 0.07	0.3092
Hemoglobin (g/dL)	13.10 ± 0.26	13.25 ± 0.19	0.6663	13.31 ± 0.19	12.82 ± 0.19	0.0848
Hematocrit (%)	39.13 ± 1.13	39.33 ± 0.25	0.8151	39.71 ± 0.65	38.43 ± 0.51	0.1268
RDW (%)	14.60 ± 0.90	14.37 ± 0.28	0.7559	14.73 ± 0.24	14.86 ± 0.19	0.6736
WBC (×10^3^/mL)	3.45 ± 0.20	8.23 ± 1.43	**0.0449**	3.58 ± 0.10	6.58 ± 0.25	**<0.0001**
Neutrophils (×10^3^/mL)	1.04 ± 0.22	4.10 ± 0.96	0.0671	1.13 ± 0.06	2.79 ± 0.19	**<0.0001**
Eosinophils (×10^3^/mL)	0.17 ± 0.05	0.54 ± 0.11	0.0655	0.23 ± 0.03	0.43 ± 0.05	**0.0059**
Basophils (×10^3^/mL)	0.04 ± 0.02	0.08 ± 0.01	0.2062	0.06 ± 0.007	0.07 ± 0.004	0.1333
Lymphocytes (×10^3^/mL)	1.91 ± 0.08	2.92 ± 0.28	**0.0461**	1.83 ± 0.06	2.85 ± 0.15	**<0.0001**
Monocytes (×10^3^/mL)	0.28 ± 0.01	0.59 ± 0.11	0.0944	0.33 ± 0.02	0.42 ± 0.02	**0.0024**
Platelets (×10^3^/mL)	233.67 ± 15.34	299.83 ± 38.24	0.2828	263.76 ± 11.62	304.96 ± 16.96	0.0811
Uric acid (mg/dL)	3.267 ± 1.007	4.133 ± 0.5989	0.1667	3.92 ± 0.99	4.31 ± 0.73	0.1194
Total cholesterol (mg/dL)	169.7 ± 17.62	204.5 ± 82.21	0.6190	172.6 ± 40.27	166.00 ± 34.64	0.4639
HDL-c (mg/dL)	39.00 ± 3.606	40.67 ± 7.528	>0.9999	41.35 ± 10.85	42.38 ± 10.17	0.6443
LDL-c (mg/dL)	111.8 ± 20.44	146.1 ± 75.91	0.6190	113.30 ± 31.73	108.40 ± 30.32	0.5345
Triglycerides (mg/dL)	94.33 ± 77.73	88.83 ± 48.80	0.7619	89.88 ± 49.42	76.08 ± 30.14	0.5588
Creatinine (mg/dL)	0.5167 ± 0.1305	0.6000 ± 0.1161	0.5000	0.76 ± 0.28	0.70 ± 0.176	0.9265
Free iron (mg/dL)	91.00 ± 22.61	80.33 ± 18.70	0.5476	98.82 ± 37.05	83.85 ± 36.84	0.1965
Glucose (mg/dL)	97.00 ± 9.539	100.7 ± 9.092	0.7619	91.24 ± 11.13	97.69 ± 9.35	0.0805
Urea (mg/dL)	29.67 ± 10.07	22.00 ± 6.870	0.4286	23.71 ± 7.69	22.42 ± 6.78	0.7629

SD: standard deviation; RBC: red blood cell; RDW: red cell distribution width; WBC: white blood cell; HDL-c: high-density lipoproteins of cholesterol; LDL-c: low-density lipoproteins of cholesterol.

**Table 3:** Association of hematological parameters with variant allele of polymorphisms in *MPO -463G>A* in both leukopenic and control children.

Hematological parameters	Presence of the *MPO* variant allele A		*p*
	Leukopenic (17)	Controls (34)	
	Mean ± SD	Mean ± SD	
RBC (×10^6^/mL)	4.67 ± 0.07	4.70 ± 0.04	0.721
Hemoglobin (g/dL)	13.17 ± 0.21	12.96 ± 0.11	0.344
Hematocrit (%)	39.36 ± 0.57	38.76 ± 0.33	0.335
RDW (%)	14.81 ± 0.23	14.95 ± 0.15	0.617
WBC (×10^3^/mL)	3.69 ± 0.07	6.96 ± 0.28	**<0.0001**
Neutrophils (×10^3^/mL)	1.17 ± 0.07	3.03 ± 0.21	**<0.0001**
Eosinophils (×10^3^/mL)	0.27 ± 0.04	0.50 ± 0.07	**0.032**
Basophils (×10^3^/mL)	0.05 ± 0.007	0.08 ± 0.005	**0.002**
Lymphocytes (×10^3^/mL)	1.87 ± 0.06	2.86 ± 0.12	**<0.0001**
Monocytes (×10^3^/mL)	0.34 ± 0.02	0.48 ± 0.02	**0.0007**
Platelets (×10^3^/mL)	247.18 ± 11.12	318.56 ± 15.55	**0.004**
Uric acid (mg/dL)	4.035 ± 1.001	4.282 ± 0.7505	0.3275
Total cholesterol (mg/dL)	158.6 ± 34.21	178.0 ± 44.54	0.3642
HDL-c (mg/dL)	39.94 ± 11.66	43.29 ± 8.476	0.1023
LDL-c (mg/dL)	103.1 ± 28.83	119.5 ± 41.59	0.4913
Triglycerides (mg/dL)	78.12 ± 42.23	76.15 ± 31.95	0.6958
Creatinine (mg/dL)	0.7853 ± 0.354	0.6503 ± 0.177	0.1614
Free iron (mg/dL)	107.6 ± 37.59	87.15 ± 38.22	**0.018**
Glucose (mg/dL)	94.76 ± 9.291	96.97 ± 9.233	0.3229
Urea (mg/dL)	23.12 ± 6.790	23.15 ± 6.378	0.7776

SD: standard deviation; RBC: red blood cell; RDW: red cell distribution width; WBC: white blood cell; HDL-c: high-density lipoproteins of cholesterol; LDL-c: low-density lipoproteins of cholesterol.

**Table 4:** Association of hematological parameters with variant allele of polymorphisms in *GSTT1* and *GSTM1* in both leukopenic and control children.

Hematological parameters	*GSTT1* null			*GSTM1* null		
	Leukopenic (4)	Controls (15)	*p*	Leukopenic (11)	Controls (20)	*p*
	Mean ± SD	Mean ± SD		Mean ± SD	Mean ± SD	
RBC (×10^6^/mL)	4.53 ± 0.13	4.79 ± 0.08	0.1410	4.70 ± 0.09	4.65 ± 0.08	0.7101
Hemoglobin (g/dL)	12.88 ± 0.43	13.12 ± 0.20	0.5956	13.20 ± 0.14	12.83 ± 0.18	0.1791
Hematocrit (%)	38.38 ± 1.16	39.08 ± 0.61	0.6032	39.53 ± 0.48	38.21 ± 0.54	0.1152
RDW (%)	14.30 ± 0.77	15.25 ± 0.24	0.1337	14.75 ± 0.33	14.75 ± 0.19	0.9898
WBC (×10^3^/mL)	3.70 ± 0.12	7.42 ± 0.52	**0.0021**	3.69 ± 0.06	7.11 ± 0.39	**<0.0001**
Neutrophils (×10^3^/mL)	1.17 ± 0.07	3.22 ± 0.34	**0.0081**	1.27 ± 0.09	3.34 ± 0.33	**<0.0001**
Eosinophils (×10^3^/mL)	0.23 ± 0.08	0.64 ± 0.14	0.1556	0.21 ± 0.04	0.42 ± 0.08	0.0618
Basophils (×10^3^/mL)	0.03 ± 0.01	0.10 ± 0.008	**0.0027**	0.05 ± 0.01	0.08 ± 0.007	**0.0449**
Lymphocytes (×10^3^/mL)	1.90 ± 0.09	2.93 ± 0.19	**0.0142**	1.84 ± 0.09	2.79 ± 0.14	**<0.0001**
Monocytes (×10^3^/mL)	0.37 ± 0.06	0.52 ± 0.05	0.1789	0.33 ± 0.02	0.48 ± 0.03	**0.0014**
Platelets (×10^3^/mL)	264.75 ± 18.75	279.67 ± 13.98	0.6141	287.45 ± 16.97	271.27 ± 12.10	0.5175
Uric acid (mg/dL)	4.45 ± 0.99	4.02 ± 0.65	0.5139	4.39 ± 0.91	4.25 ± 0.84	0.7525
Total cholesterol (mg/dL)	152.30 ± 42.53	172.10 ± 31.22	0.9801	180.40 ± 43.91	164.80 ± 34.72	0.0967
HDL-c (mg/dL)	39.25 ± 7.14	43.60 ± 6.30	0.2131	41.09 ± 10.35	44.30 ± 9.75	0.4323
LDL-c (mg/dL)	101.10 ± 37.82	114.90 ± 31.31	0.8989	119.20 ± 34.15	106.20 ± 29.32	0.1202
Triglycerides (mg/dL)	59.75 ± 19.52	68.33 ± 22.67	0.7500	100.40 ± 52.68	71.45 ± 37.12	0.0927
Creatinine (mg/dL)	0.65 ± 0.22	0.65 ± 0.16	0.9900	0.79 ± 0.35	0.61 ± 0.16	0.1790
Free iron (mg/dL)	88.75 ± 41.77	71.07 ± 22.19	0.3965	85.45 ± 41.90	86.20 ± 33.66	0.7069
Glucose (mg/dL)	93.25 ± 10.28	96.27 ± 6.47	0.5175	90.73 ± 9.56	97.50 ± 8.49	0.0882
Urea (mg/dL)	22.75 ± 4.11	24.47 ± 6.59	0.6068	24.73 ± 8.41	22.35 ± 4.52	0.7516

SD: standard deviation; RBC: red blood cell; RDW: red cell distribution width; WBC: white blood cell; HDL-c: high-density lipoproteins of cholesterol; LDL-c: low-density lipoproteins of cholesterol.

**Table 5:** Multivariate analysis associating gene polymorphisms related to xenobiotic metabolism among children with leukopenia.

Variables	*Β*	SD	*t*	*p*	*p* of model
Model 1					
Female	−0.444	0.178	−2.499	0.019	0.049
Age	−0.193	0.168	−1.153	0.258	
*NQO1 609C>T*	0.208	0.165	1.260	0.218	
Model 2					
Female	−0.536	0.189	−2.842	0.008	0.037
Age	−0.207	0.166	−1.243	0.224	
*GSTM1*	−0.273	0.184	1.480	0.150	
Model 3					
Female	−0.537	0.188	−2.859	0.008	0.048
Age	−0.208	0.165	−1.255	0.220	
*GSTM1*	0.252	0.185	1.363	0.184	
*NQO1 609C>T*	0.185	0.163	1.132	0.267	
